# Multimodal Prediction of Psychosis in the Prospective MoBa Birth Cohort

**DOI:** 10.21203/rs.3.rs-6783339/v1

**Published:** 2025-06-20

**Authors:** Viktoria Birkenæs, Pravesh Parekh, Alexey Shadrin, Piotr Jaholkowski, Lars A. R. Ystaas, Carolina Makowski, Nora R. Bakken, Espen Hagen, Evgeniia Frei, Dominic Oliver, Paolo Fusar-Poli, Anders Dale, John P. John, Alexandra Havdahl, Ida E. Sønderby, Ole A. Andreassen

**Affiliations:** Oslo University Hospital; Oslo University Hospital; Oslo University Hospital; Oslo University Hospital; Oslo University Hospital; University of California, San Diego; Oslo University Hospital; Oslo University Hospital; Oslo University Hospital; University of Oxford; King’s College London; University of California, San Diego; National Institute of Mental Health and Neurosciences (NIMHANS); Norwegian Institute of Public Health; Oslo University Hospital; Oslo University Hospital

**Keywords:** MoBa, psychosis, prediction, machine learning

## Abstract

There is a need for improved early psychosis detection beyond the traditional clinical high-risk strategy. Using the Norwegian Mother, Father and Child cohort study, we examined the predictive ability of self-reported psychotic experiences (Community Assessment of Psychic Experiences; CAPE) at age 14, in addition to general mental health factors, parent and childhood psychiatric diagnoses, schizophrenia polygenic risk scores, and birth-related factors, to predict subsequent psychosis onset using three machine learning approaches for imbalanced data. We explored also a multimodal prediction framework. For unimodal classification, we observed best balanced accuracies with general mental health factors (67.27 ± 1.76%), and CAPE (65.95 ± 1.09%). Multimodal models improved classification accuracy (68.38 ± 2.16%). With validation and additional model refinement, these features may be useful for initial screening within clinical stepped assessment frameworks.

## Introduction

Psychosis typically emerges in early adulthood,^1^ but attenuated or prodromal symptoms often appear during adolescence.^2^ Longer duration of untreated psychosis is associated with poorer outcomes^3^ and substantial subjective burden.^4^ Therefore, improving early detection of psychosis is vital.^5^ Most psychosis detection research has focused on samples with help-seeking individuals at clinical high-risk for psychosis (CHR-P).^6–8^ However, many at-risk individuals avoid seeking help,^7^ and only a small percentage (> 15%) of people treated for a first episode psychosis are identified in CHR-P services,^9–11^ despite 78% experiencing prodromal symptoms prior to their first episode.^12^ Additionally, in help-seeking individuals who do not meet the criteria for CHR-P, ~1.6% transition to psychosis within 38 months.^13^ New approaches for early detection beyond the current CHR-P framework may help identify more individuals who develop psychosis.^14,15^ Such prediction approaches can take advantage of several putative predictors, including psychotic experiences,^16^ general adolescent mental health and functioning,^17^ genetic risk of schizophrenia,^18^ parental^19,20^ and childhood psychiatric diagnoses,^21^ and pre- and perinatal factors.^22,23^

CHR-P status is defined based on attenuated psychotic symptoms, genetic risk or family history of psychosis combined with a marked functional decline^7,14^ and are assessed through clinical interviews such as the CAARMS.^24–27^ These interviews have high prognostic accuracy but limited group-level predictions.^28^ Transition rates from a CHR-P state to psychosis vary with time (from 20% at two years to 35% at ten years)^29^ and across subgroups,^29,30^ with the highest risk of transition in individuals with brief psychotic episodes.^31,32,32–37^ In CHR-P samples from specialised clinics, internally validated psychosis prediction models show wide-ranging performances. Average sensitivities and specificities are65.9% and 68% for models using clinical features (e.g., psychotic symptoms, family history, and premorbid functioning), 69.6% and 84.5% for biomarkers (e.g., genetic risk scores, immune markers, and brain imaging), 71.8% and 81% for cognitive features (e.g., verbal learning and processing speed), and 52.4% and 76.8% for environmental predictors (e.g., urbanicity, childhood trauma, and drug-use).^7,15,38^ CHR-P multimodal prediction models show average sensitivities of 76.6% and specificities of 74.4%.^7,15,38^

Fewer studies have investigated psychosis prediction in population samples. One example is Sullivan et al.^39^ who used used data from electronic health records (non-psychotic diagnoses, medication, number of consultations, age, gender, social deprivation, geographical location, and ethnicity), yielding a sensitivity of 71% and specificity of 84% using Cox regression in predicting the onset of psychosis over six years. External validation of the model showed a sensitivity of 65.6% and specificity of 86.6%.^40^

Psychosis has a multifactorial aetiology,^41,42^ and improving early diagnosis will likely require multimodal prediction. Some sources of data, like biomarkers, are costly and usually not accessible during the initial stages of healthcare. Self-report tools are a promising strategy for early detection as they allow for time-effective screening to identify individuals who might require further clinical evaluation in specialised clinics. There has been a growing interest in psychotic experiences (PEs): sub-clinical hallucinations and delusions. These appear in general populations at a much higher frequency than psychotic disorders^43,44^ and are associated with an increased risk of conversion to psychosis.^18,26,27^ Evidence from longitudinal cohorts shows strong associations between reports of PEs in childhood and adolescence and adult-onset psychosis.^45–50^ We previously observed a relationship between PEs reported at age 14 and subsequent psychotic disorders^51^ in the Norwegian Mother, Father, and Child cohort study (MoBa).^52,53^ Therefore, self-reported PEs could be valuable in psychosis screening and early psychosis detection outside of specialised clinics when combined with other predictors.

There are methodological challenges to predicting psychosis which impact the reproducibility of models. First, there is a trade-off between simple and complex modelling approaches. Simple models (i.e., logistic regression) are transparent and easily interpretable while complex machine learning approaches capture nuanced relationships but are more sensitive to overfitting.^54^ A common source of overfitting is improper separation between training and test data.^55^ Samples with substantial heterogeneity also complicate the development of generalisable models.^56^ In population samples, the low lifetime prevalence of psychosis (~3%)^57^ creates a substantial imbalance between the number of cases and controls, making it difficult to predict more than just the majority class. Together, these challenges undermine the reproducibility – and potential future clinical value – of psychosis detection models and should be addressed.^54^

In the current study, we explored the predictive performance of the Community Assessment of Psychic Experiences (CAPE) at age 14 in predicting a subsequent psychosis diagnosis, compared to other putative predictors: general adolescent mental health factors,^17^ genetic risk of schizophrenia,^18^ parental^19,20^ and childhood psychiatric diagnoses,^21^ and pre- and perinatal factors^22,23^ in the population-based prospective MoBa cohort. In addition, we explored multimodal prediction of psychosis by combining CAPE with these four other predictors.

## Results

We included data from the prospective Norwegian Mother, Father and Child Study creating five separate predictor groups: 1) self-reported psychotic experiences (as measured with the Community Assessment of Psychic Experiences [CAPE]), 2) self-reported general mental health factors at age 14, 3) registry parent and childhood psychiatric diagnoses, 4) schizophrenia polygenic risk scores (PRS), and 5) registry birth-related risk factors. Using these features individually, we modelled the onset of psychotic diagnoses (ICD-10^58^ and ICPC^59^) after the age of 14. We evaluated three machine learning approaches (undersampling, oversampling, and cost-sensitive learning) using 50 repeats of 5-fold stratified cross-validation. For multimodal prediction using all five predictors, we created 10 combinations of two modalities at a time, as well as one model with all five predictor groups.

The participants in the psychotic experiences predictor group sample (*n* = 22,952; 47 cases) were 18.64 ± 1.52 years old (range: 15–21 years; 53.5% female). In the general mental health predictor group (*n* = 23,512; 48 cases), participants were 18.64 ± 1.52 years old (range: 15–21 years; 53.9% female); in the schizophrenia PRS group (*n* = 73,335; 242 cases), participants were 18.69 ± 2.19 years old (range: 15–24 years; 48.7% female); in the parent and childhood psychiatric diagnoses group (*n* = 108,877; 378 cases), participants were 18.62 ± 2.20 years old (range: 15–24 years; 48.9% female); and in the birth-related risk factor group (*n* = 106,937; 375 cases), participants were 18.61 ± 2.20 years old (range: 15–24 years; 48.8% female).

**Figure 1:**
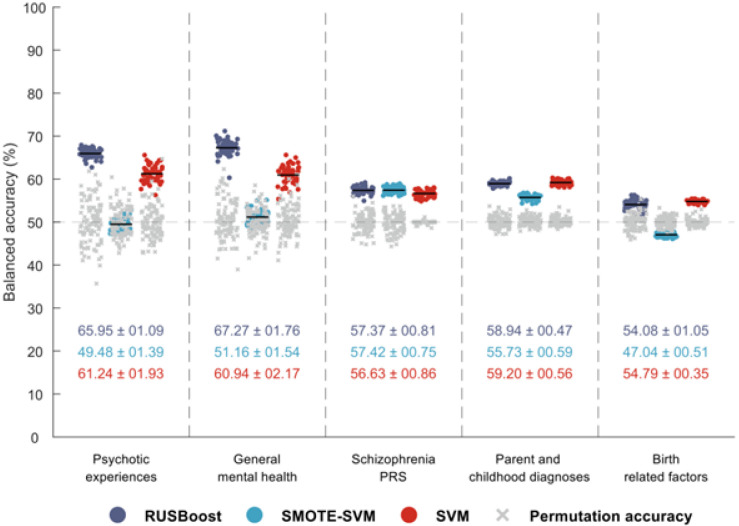
Balanced accuracies of the five unimodal prediction group models

General mental health predictors with the undersampling approach showed the overall highest balanced accuracy (BAC) of 67.27 ± 1.76 (range: 60.31–71.16) across all predictor groups and machine learning approaches. This was closely followed by the psychotic experiences predictor group with a BAC of 65.95 ± 1.09 (range: 62.73–67.91; Figure 1). Generally undersampling outperformed the oversampling and cost-sensitive learning approaches, except in the case of schizophrenia PRS, where undersampling did not clearly outperform oversampling, and in the case of birth factor features where the cost-sensitive SVM slightly outperformed undersampling (54.79 ± 0.35 compared to 54.08 ± 1.05). We observed the highest sensitivity with undersampling for schizophrenia PRS (62.54 ± 1.88). Figure 1 presents the balanced accuracies of all three classification approaches for all predictor groups, while Table 1 also presents sensitivities and specificities. Supplementary Table S1 provides detailed performance results.

Overall, there were more individuals correctly classified between the ages of 15 to 17 compared to later ages (Supplementary Figures S1-S5). In comparison, schizophrenia PRS and birth-related factors appeared to predict equally well throughout the age range. From our sensitivity analysis, we observed slightly higher accuracies when including sex assigned at birth in the general mental health features: from 66.92 ± 1.55 without to 67.27 ± 1.76 with sex using undersampling (oversampling: 49.80 ± 1.36 without sex; 51.16 ± 1.54 with sex; cost-sensitive learning: 61.42 ± 2.38 without sex; 60.94 ± 2.17 with sex).

The performance of each predictor group using three machine learning approaches: Undersampling (RUSBoost: random undersampling boosting^60^), oversampling (SMOTE: synthetic minority oversampling^61^), and cost-sensitive learning with SVM (support vector machine). See supplementary Table S1 for detailed performance metrics.

For multimodal prediction, we tested all possible pairs of the predictor groups (10 combinations) using the best performing machine learning approach (undersampling). This was done to mitigate multiple testing issues. We subset the data to only include participants with complete data for both predictor groups in each pair. We then trained unimodal and combined bimodal models for each pair of predictors, and then a final model combining the three highest-performing predictor groups.

**Figure 2:**
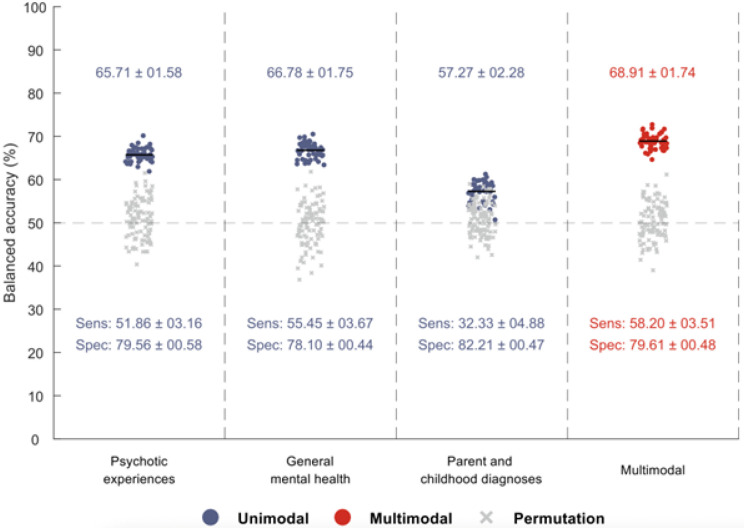
Three individual predictor groups and a combined model

The most optimal prediction model combined three predictor groups: psychotic experiences (CAPE), general mental health factors, and parent and childhood diagnoses, yielding a BAC of 68.91 ± 1.74 (range: 64.63–72.74). This trimodal approach consistently outperformed all unimodal approaches, surpassing unimodal CAPE 86% of the time (43 of 50 iterations), general mental health factors 86% of the time (44 of 50 iterations), and parent and childhood diagnoses 100% of the time (50 of 50 iterations). Figure 2 presents these results, while Supplementary Table S3 gives detailed estimates for all combinations of models.

Among the individual predictor contributions, the combinations of psychotic experiences and general mental health factors emerged as the strongest bimodal pairing, with a BAC of 68.38 ± 2.16 (range: 62.72–73.83). This bimodal model outperformed unimodal CAPE 88% of the time (44 of 50 times) and unimodal general mental health factors 70% of the time (35 of 50 times).

The additional bimodal combinations also showed improvements. General mental health factors combined with either pre- and perinatal factors (BAC: 67.59 ± 1.67 [range: 63.64–71.32]) or parent and childhood diagnoses (BAC: 67.32 ± 1.75 [range: 64.31–71.53]) demonstrated higher accuracies than their unimodal counterparts. Similarly, schizophrenia PRS showed improved performance when combined with pre- and perinatal factors (BAC: 58.41 ± 0.86 [range: 55.43–60.24]) or parent and childhood diagnoses (BAC: 58.89 ± 0.69 [range: 57.36–60.02]), though overall performance remained lower than other predictor groups.

Running the individual models on a subset of the sample containing participants with complete data (from all sources) gave results comparable to the ones observed for the non-overlapping datasets, although with slightly lower estimates (Supplementary Table S3).

Comparison of the prediction performance of the individual models and a combined model consisting of all three predictors: psychotic experiences, adolescent general mental health symptoms, and parent’s and children’s mental health diagnoses. Analyses were conducted using undersampling (RUSBoost).

## Discussion

The results of this study suggest that adolescent psychotic experiences – as measured with the CAPE-9 questionnaire – can predict subsequent psychosis onset in individuals from a population-based sample. We also observed performance differences the three machine learning approaches we used to handle class imbalance: under- and oversampling and cost-sensitive learning. In pairwise combinations of the five predictor groups (psychotic experiences, general mental health factors, parent and childhood psychiatric diagnoses, schizophrenia PRS, and pre-and perinatal risk factors), the prediction accuracy increased. A multimodal approach combining adolescent psychotic experiences with general mental health and parent and childhood diagnoses resulted in the highest average performance.

In this population-based sample, adolescent general mental health factors and psychotic experiences measured with CAPE were the most effective predictors for identifying individuals who develop psychosis. Both measures were better at predicting psychosis development within the near future (average time to follow-up: 3.91 ± 2.21), correctly classifying more individuals who develop psychosis between the ages of 15 to 17 compared to later (Supplementary figures S1-S5). In comparison, schizophrenia PRS and birth-related factors appeared to predict equally well throughout the age range (15 to 24 years). Adolescent psychotic experiences and general mental health factors likely represent more immediate, state-dependent risk markers whose predictive power diminishes over time. In contrast, the diagnostic, genetic, and birth-related predictors seem to be stable trait-like predisposing factors that result in enduring risk pathways, regardless of age.

The general adolescent mental health predictors performed somewhat better than CAPE. This predictor group included self-reported traumatic experiences and drug use, as well as social competence, problems in relationships with peers and parents, and social anxiety. These factors likely reflect key aspects in the development of psychosis, particularly accumulating challenges with and withdrawal from social interactions.^62^ While CAPE assesses positive psychotic experiences (i.e., hallucinations and delusions), it does not capture negative symptomatology, such as social withdrawal. Therefore, the adolescent general mental health predictors likely encompass a wider range of key psychosis predictors.

We observed higher average accuracies when combining adolescent psychotic experiences and general mental health and an additional small increase when adding parental and childhood diagnoses, supporting the use of a multimodal approach. A potential reason for the modest performances is that early symptoms preceding psychosis are typically non-specific.^63,64^ As many experience heterogeneous illness trajectories, some researchers argue for the existence of pluripotent mental health risk states.^65^ When predicting adult mental health outcomes from early adolescence, a transdiagnostic approach may be more beneficial, where – regardless of specific diagnoses – individuals who are likely to develop any mental condition will benefit from closer monitoring and follow-up. Because of the restricted samples and exploratory nature of the analyses, we cannot conclude based on the current results. Nonetheless, our preliminary findings support the potential value of multimodal psychosis prediction models in population-based samples.

Several studies have used psychotic experiences to predict psychosis onset in non-enriched samples, reporting somewhat higher estimates than our findings.^48,66–68^ We also lacked the sample size necessary to create an independent validation set. However, common methodological issues in psychosis prediction research, such as inadequate training and test set separation and improper handling of class imbalance, can artificially inflate prediction estimates.^7,38,56^ We tried to mitigate risks of overfitting by handling imbalanced classes using three machine learning approaches and family-based cross-validation to account for sample relatedness. While further model refinement is necessary, our preliminary findings support that models based on simple, cost-effective screening measures can be utilised for psychosis prediction models in population-based samples.

Across the three machine learning approaches, random undersampling resulted in higher balanced accuracies. Whether an approach to handle imbalance outperforms other methods will change depending on the size and distribution of the sample. Thus, there is no consensus on an optimal approach.^69–72^ For instance, cost-sensitive learning might be more robust in larger datasets (*n* >10,000), as compared to over- or undersampling, but performs poorly on small samples.^72^ Moreover, it has been proposed that under- and oversampling may lead models to overestimate instances of the minority class.^73^ Further, imbalance correction reduces the clinical use of a model, and some suggest it is better to adjust the probability threshold.^73^ Calibration of these probabilities requires additional cross-validation (e.g., Platt scaling^74^), and given the relatively small number of cases in our sample, we were not able to create calibration or decision curves, which are used to evaluate a model’s clinical value and potential cost-benefit decisions.^75^ In future work, when more data are available, incorporating probability-based scores like the Brier score^76^ would likely result in better-calibrated models.

Except for schizophrenia PRS and parent and childhood psychiatric diagnoses, oversampling via SMOTE (Synthetic Minority Oversampling Technique^61^) followed by SVM (support vector machine) classification generally resulted in chance-level performance. Oversampling works by creating synthetic datapoints for the minority class in the training sample. With ordinal features (such as the general mental health and CAPE predictor groups), oversampling sometimes led to feature values for the cases in the training set that did not match the distribution of values in the test set. Therefore, the trained model would not generalise to the test set, leading to chance-level performances. An example of this phenomenon is illustrated in Supplementary Figure S6. This was not the case for the schizophrenia PRS or parent and childhood psychiatric diagnoses predictor groups as the features were either all continuous (PRS) or all categorical. In these scenarios, the oversampled training distribution did not deviate substantially from the test set distribution.

Our study has notable strengths. We investigated a range of psychosis risk features (i.e., mental health self-reports, registry data, and genetics) selected based on prior evidence to optimise the accuracy and robustness of our models. Additionally, the prospective population-level data allowed us to broaden the scope of prediction beyond traditional high-risk samples. We applied three machine learning approaches with a comprehensive analysis of different algorithms in situations of severe class imbalance. Moreover, we took specific steps to avoid data leakage, performing all preprocessing, sampling, and standardization on pre-selected features within family-based cross-validation (see Supplementary Note 1).

Our analyses also have limitations. Participants were between 15 and 24 years old with an average follow up time of 4 years. Thus, more individuals from the MoBa offspring generation will develop psychosis in the upcoming years, likely resulting in false negatives in the current estimates. Due to the limited number of cases, we were unable to split our data into an independent hold-out sample and internally validate our models. Another issue is non-representativeness and selection bias, which can affect the clinical use of prediction models.^77^ Compared with the total dataset, the adolescents who responded to the latest wave of questionnaires had older and more well-educated parents^78,79^ and had a lower prevalence of severe mental disorders.^51,80^

Our findings demonstrate that self-reported adolescent psychotic experiences and general mental health factors show promise for predicting transition to psychosis in population-based samples. These are mental health measures are easily administered, and their superior performance suggest their potential value in early screening. Additionally, our findings also provide insights into the use of sampling and cost-sensitive learning strategies to severe class imbalance. We also found support for higher average accuracies with multimodal prediction. Validation in independent samples with larger number of cases will be essential to establish the true performance of our multimodal approach.

With appropriate validation and refinement, these models could form the foundation for improving psychosis detection beyond traditional high-risk approaches. A stepwise screening framework – where accessible mental health assessments provide initial risk stratification followed by comprehensive clinical evaluation for higher-risk individuals – represents a promising avenue for early identification and intervention in psychosis.

## Methods

### Data and participants

The Norwegian Mother, Father, and Child Cohort Study (MoBa) is a population-based pregnancy cohort study conducted by the Norwegian Institute of Public Health.^52,53^ Participants were recruited across Norway from 1999 to 2008. The women consented to participation in 41% of the invited pregnancies. The complete cohort includes approximately 114,500 children, 95,200 mothers and 75,200 fathers. The current study is based on version 12 of the quality-assured data files released for research in 2019. The establishment of MoBa and initial data collection were based on a license from the Norwegian Data Protection Agency and approval from The Regional Committees for Medical and Health Research Ethics. The MoBa cohort is currently regulated by the Norwegian Health Registry Act. The current study was approved by The Regional Committees for Medical and Health

Research Ethics (2016/1226/REK sør-øst C). For this work, we restricted the sample to participants who had not withdrawn their consent as of the 1^st^ of November 2024. We have adhered to the reporting guidelines for cohort (STROBE^81^) and prediction studies (TRIPOD^82^). The guideline checklists are appended in the supplementary materials.

### Exclusion criteria

We excluded individuals who withdrew their consent before the latest data update (2024–11-1; *n* = 4,354), children whose parents did not have a specified national ID number (*n* = 17), individuals without information about birth year from the Norwegian Medical Birth Registry (MBRN; *n* = 106), individuals with mismatched sex information between the birth registry and genetic data (*n* = 3), and children diagnosed with a psychotic disorder six months our diagnostic cut-off as described below (*n* = 87).

### Outcome

We defined an individual as having a psychotic disorder if they received any ICD-10^58^ F2 chapter diagnoses (F20, F21, F22, F23, F24, F25, F28, and F29) or P72 (schizophrenia) or P98 (psychosis not otherwise specified) ICPC^59^ codes after a minimum of six months following the last questionnaire response (see below) or, for non-responders, six months after turning 14 years old. As CAPE was our main predictor of interest, we used this questionnaire to define the cut-off. All individuals diagnosed with psychosis six months or more after responding to CAPE were defined as a case, while those diagnosed prior to this were removed from analyses. The reason for using this six-month margin was to avoid including individuals who are already in the prodromal phase of psychosis. For those who did not respond to CAPE, case status was defined based on whether they were diagnosed with psychosis six months or more after turning 14 years, the average age at CAPE response. Anyone diagnosed with psychosis before this was excluded. The month when participants turned 14 years old was calculated using birth year and month from the MBRN. Supplementary Figure S7 gives an overview of the age at which participants were first diagnosed with a psychotic disorder in the complete dataset. Supplementary Figure S8 shows the ages of the participants (cases and controls) in days and years when the predictors are being considered based on the predictor cutoffs.

We combined diagnostic information from two sources: the Norwegian Patient Registry (NPR; diagnostic information available from 2008 to December 2023) and the Norwegian Control and Payment of Health Reimbursements Database (KUHR; information available from 2006 to October 2023). The NPR contains ICD-10 diagnoses from hospitals, outpatient clinics, or contract specialists in Norway. The KUHR contains reimbursement information from all public health services and includes ICD-10 diagnoses and ICPC codes (versions 1, 2 and 2b) in Norway. The average time between the predictor cut-off and the latest diagnostic update was 3.91 ± 2.21 years.

### Predictors

Predictor variables were selected among factors previously reported to be associated with risk of psychosis and available in the MoBa cohort – genetic risk,^18^ pre- and perinatal exposures,^22,23^ parental^19,20^ and premorbid non-psychotic psychiatric diagnoses,^21^ childhood adversity, and impaired social and general functioning.^17^ We grouped these 164 predictors into five groups related to: adolescent psychotic experiences, adolescent general mental health factors, schizophrenia polygenic risk scores, parent and childhood psychiatric diagnoses, and pre-and perinatal risk factors. As CAPE was administered the year participants turned 14 years old, we restricted all predictors to only include data collected until CAPE questionnaire response or the age of 14 (for non-responders). Table 2 presents the predictor groups and their sample sizes. A detailed overview of variables included under each predictor is reported in Supplementary Table S3. A brief description of each predictor group follows.

### Psychotic experiences

Between 2017 and 2023, 25,154 MoBa children (aged ~14 years) responded to a health and lifestyle questionnaire (Q-14). The Q-14 questionnaire included CAPE-16, which comprises CAPE-15^83–85^ and an additional item (*delusions of reference*) from CAPE-9.^80,86^ Each item is rated on a 4-point scale for both frequency of psychotic experiences and related distress (“never”, “sometimes”, “often”, “nearly always”). Both CAPE-9 and CAPE-16 have demonstrated good psychometric properties in MoBa adolescents and fathers.^51,80^ As data collection was initiated using the shorter CAPE-9, 9% (*n* = 2,264) of the sample did not respond to CAPE-16. Hence, in the current analyses, we used CAPE-9 (*n* = 22,953) frequency and distress scales, their interaction term, and individual items as predictors.

### Adolescent general mental health

In this predictor group, we included variables from the Q-14 questionnaire related to general mental health, encompassing self-reported adverse life events, current drug use and mental health symptoms, and social functioning.

### Schizophrenia polygenic risk score

Blood samples were collected from umbilical cords after birth. Samples were processed and stored, and DNA was extracted and genotyped using standard methods.^87^ We used the quality-controlled genetic data^88^ from 73,093 children who had not withdrawn their consent, did not have ambiguous sex information, and who had available information on birth year. We calculated PRS with PRSice2 version 2.3.5^89^ based on genome-wide association study summary statistics from the Psychiatric Genomics Consortium for schizophrenia^90^ using eleven *p*-value thresholds: 1, 0.5, 0.1, 0.05, 0.01, 0.001, 1e-04, 1e-05, 1e-06, 1e-07, 5e-08. We used these eleven PRSs as predictors.

### Parent and childhood psychiatric diagnoses

We included childhood psychiatric diagnoses of participants as well as adult psychiatric diagnoses from both parents. Specifically, we included diagnoses of substance use disorders (F10–19), bipolar disorder (F31), depressive episodes (F32) or recurrent depression (F33) without psychotic features, any anxiety and stress-related disorders (F40–43), eating disorders (F50), personality disorders (F60), autism spectrum disorder (F84), and ADHD (F90). All diagnostic information was derived from the diagnostic registries (NPR and KUHR). To prevent overlap between the onset of psychosis and the time of the diagnostic predictors, we only included diagnostic predictors obtained six months prior to CAPE response or, for those who did not respond to CAPE, the date when they turned 14 years old.

### Pre- and perinatal factors

We included variables characterising the pregnancy, delivery, and health of the child and mother at birth using information from questionnaires answered by the mothers during pregnancy (weeks 15, 22, and 30) and from the Norwegian Medical Birth Registry. These included variables related to maternal infections during pregnancy, foetal birth weight, and head circumference (see Supplementary Table S3 for the full list). For head circumference and weight, we excluded extreme values (beyond the 5^th^ and 95^th^ percentile) using the growth references for Norwegian infants.^91^

### Machine learning

To handle class imbalance, we fit models for each of the five predictor groups using three machine learning approaches (undersampling, oversampling, and cost-sensitive learning) within the family-based stratified cross validation strategy. What follows are the details of this machine learning procedure.

### Family-based cross validation

**Figure 3:**
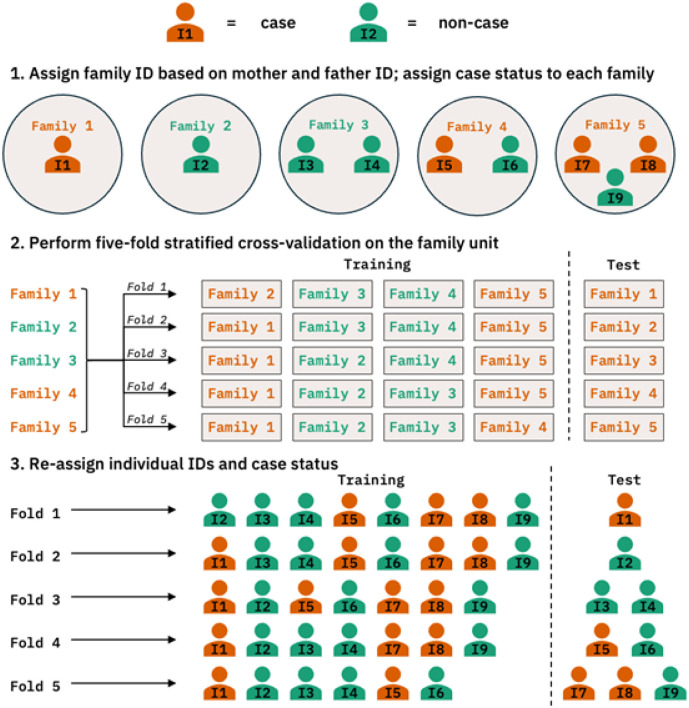
Steps for handling siblings within a stratified cross-validation framework *Note*. To make sure that siblings were always either in the training or test sets, we took a family-based sampling approach when dividing the data for cross-validation. 1) Individuals sharing the same parent IDs were defined as siblings. 2) We collapsed siblings into a single family unit with the case status being 1 if any of the siblings had a psychotic disorder. 3) We performed stratified cross-validation on the family unit, ensuring an equal number of cases and controls between training and test sets. 3) Once samples in each set were defined, we transformed the family units back into individual observations.

As the MoBa dataset includes related individuals, not all observations are independent. To ensure that siblings were always within training or test set (but never in both), we opted for a stratified sampling approach: first, we defined a family unit using the parent ID of each individual. Subjects having the same mother and/ or father were identified as siblings. Next, we collapsed siblings into a single observation with the case status being “1” if any of the siblings had a psychotic disorder. Then, we performed a family-based stratified cross-validation on the family unit. Once the samples were split between training and test sets, we expanded the family units back into individual observations. Figure 3 gives a visual description of the strategy.

### Handling imbalance

With considerably more non-psychosis individuals than psychosis cases (see Table 2), we used three machine learning approaches to deal with class imbalance: undersampling (samples from the majority class are removed or undersampled), oversampling (samples from the minority class are synthesised or oversampled), and cost-sensitive learning (machine learning parameters are modified to handle class imbalance). For undersampling, we used the random undersampling boosting (RUSBoost)^60^ classifier approach where the learning rate was set to 0.1 and the number of learning cycles to 100. For oversampling, we used the synthetic minority oversampling (SMOTE)^61^ approach followed by a support vector machine (SVM) classifier; we used SMOTE for continuous features, SMOTEN for nominal data, and SMOTENC for numerical and categorical features, as implemented in the Python imbalanced-learning library, version 3.12.3.^92^ The lambda was set to automatic. For cost-sensitive learning, we used SVM and optimised the misclassification cost using nested cross-validation.

For undersampling and oversampling, we performed 50 repeats of 5-fold cross-validation. For cost-sensitive SVM, we performed 50 repeats of 5-fold nested cross-validation (with the inner split also set to 5 folds). Within the inner cross-validation, we optimised the cost term for SVM using a grid search (with increments of 1, 2, 5, 10, 50, 100, 250, 500, 750, 1000). To determine which of the cost values were optimal, we used average balanced accuracy across the test sets in the inner cross-validation. Then, the optimal cost was used in the outer fold training set to train the classifier.

Within each cross-validation fold (both inner and outer splits), we standardised all continuous features (i.e., rescaling the features to have zero mean and unit standard deviation). The standardization was always done on the training set, and the mean and standard deviations were applied to the test set. Further, for schizophrenia PRS, we regressed the effect of the first 20 genetic principal components (PCs) from the PRS prior to using them as features. The regression coefficients were learnt from the training set and applied to the test set. To avoid data leakage, we calculated the PCs in the 1000 genome project samples^93^ using a subset of overlapping SNPs between MoBa and the 1000 genome project. We then projected the MoBa samples to these PCs. Supplementary Note 1 describes the steps we took to mitigate all potential sources of leakage. We included sex as a predictor variable in the general mental health predictor group. As sex might boost the predictive value of this predictor group relative to the other groups, we ran the same model excluding sex in a sensitivity analysis.

To assess if the model’s average performance was above chance levels, we permuted class labels and reapplied the entire pipeline 100 times. This gives us an estimate of model performance under the null (i.e., there is no association between the features and the outcome). Then, we calculated the *p*-value as the proportion of times the permuted performance was greater than or equal to the actual performance.^94^

### Post hoc exploration of multimodal prediction

We explored all paired combinations of the predictor groups (one-by-one predictor combinations). Since this is a combinatorial problem, we performed this post hoc analysis only using RUSBoost and considered two modalities at a time (10 combinations). First, we subset the data to include participants with data from both predictor groups in each pair, resulting in datasets without missingness. Next, in these restricted samples, we trained both new unimodal and bimodal models using the cross-validation approach described above. In a final model, we combined the three highest performing predictor groups. To compare multimodal vs unimodal prediction performances, we counted the number of times the multimodal BAC outperformed the unimodal BAC – we used a rule of thumb of an improvement if the multimodal BAC was higher than the unimodal BAC more than 75% times.^95^

Data cleaning and preparation were performed in RStudio, R version 4.2.3.^96^ All machine learning models were trained using MATLAB R2023a,^97^ except for SMOTE, which was implemented in the Python (v3.9.6) “imbalanced-learn” library (v0.12.3),^92^ built on “scikit-learn” (v1.5.1).^98^

## Supplementary Material

Supplementary Files

This is a list of supplementary files associated with this preprint. Click to download.

- 250530Supplement.docx

## Figures and Tables

**Table 1.** Prediction performance across five predictor groups and three classifier approaches

Classifier	Feature	*n* cases/controls	Sensitivity	Specificity	Balanced accuracy
**Undersampling**	Psychotic experiences	47/22905	52.23 ± 2.26 [45.56–57.33]	79.66 ± 0.64 [78.11–80.88]	65.95 ± 1.09 [62.73–67.91]
	General mental health	48/ 23464	56.24 ± 3.63 [41.11–64.67]	78.30 ± 0.57 [77.13–79.70]	67.27 ± 1.76 [60.31–71.16]
	SCH PRS	242/73093	62.54 ± 1.88 [57.87–67.36]	52.20 ± 0.82 [50.50–53.81]	57.37 ± 0.81 [54.93–59.20]
	Parent and childhood psychiatric diagnoses	378/108499	31.10 ± 0.97 [28.31–33.35]	86.77 ± 0.51 [85.34–87.81]	58.94 ± 0.47 [57.81–60.14]
	Birth-related factors	374/106562	44.04 ± 2.29 [38.19–48.42]	64.11 ± 1.05 [61.68–66.63]	54.08 ± 1.05 [51.67–56.26]
**Cost-sensitive**	Psychotic experiences	47/22905	32.48 ± 4.25 [25.33–40.00]	90.01 ± 1.99 [84.64–92.85]	61.24 ± 1.93 [56.25–65.56]
	General mental health	48/ 23464	36.45 ± 5.55 [21.11–49.78]	85.42 ± 3.60 [77.56–92.96]	60.94 ± 2.17 [55.36–65.61]
	SCH PRS	242/73093	41.08 ± 1.81 [36.83–43.79]	72.18 ± 0.51 [71.16–73.42]	56.63 ± 0.86 [54.85–58.02]
	Parent and childhood psychiatric diagnoses	378/108499	30.35 ± 1.28 [27.51–32.81]	88.04 ± 0.28 [87.34–88.65]	59.20 ± 0.56 [58.08–60.29]
	Birth-related factors	374/106562	28.38 ± 0.75 [26.48–29.95]	81.19 ± 0.62 [79.57–82.25]	54.79 ± 0.35 [53.95–55.42]
**Oversampling**	Psychotic experiences	47/22905	1.36 ± 1.59 [0.00–6.44]	97.60 ± 2.29 [94.06–99.56]	49.48 ± 1.39 [47.14–52.93]
	General mental health	48/ 23464	14.42 ± 3.11 [8.22–22.89]	87.90 ± 0.41 [86.99–88.80]	51.16 ± 1.54 [48.34–55.13]
	SCH PRS	242/73093	59.73 ± 1.51 [57.04–62.81]	55.10 ± 0.18 [54.66–55.57]	57.42 ± 0.75 [56.06–58.93]
	Parent and childhood psychiatric diagnoses	378/108499	25.01 ± 1.66 [21.94–28.79]	86.45 ± 1.37 [83.27–89.33]	55.73 ± 0.59 [54.25–56.79]
	Birth-related factors	374/106562	28.11 ± 1.81 [22.73–30.76]	65.97 ± 1.85 [64.99–72.40]	47.04 ± 0.51 [46.09–47.92]

SCH PRS = schizophrenia polygenic risk scores. The performance of each predictor group using three machine learning approaches: Undersampling (RUSBoost: random undersampling boosting^60^), oversampling (SMOTE: synthetic minority oversampling^61^), and cost-sensitive learning with SVM (support vector machine). See supplementary Table S1 for detailed performance metrics.

**Table 2.** Description of the five groups of psychosis predictors

Predictor groups	Description	No. of features	*n* (%) controls	*n* (%) cases
Psychotic experiences	Self-reported sub-clinical psychotic symptoms measured at age 14*	28	22,905 (99.8%)	47 (0.2%)
General mental health	Self-reported general mental health symptoms measured at age 14*	41	23,464 (99.8%)	48 (0.2%)
Schizophrenia polygenic risk scores	Polygenic risk scores for schizophrenia	5	73,093 (99.7%)	242 (0.3%)
Parent and childhood psychiatric diagnoses	Specialist mental healthcare diagnoses from parents and children before the youth turned 14 years	32	108,499 (99.7%)	378 (0.3%)
Birth-related factors	Pre- and perinatal factors associated with psychosis risk	50	106,562 (99.6%)	375 (0.4%)

* To avoid temporal overlap, we set a six-month difference between when the predictor variables and the outcome were collected.

## Data Availability

The consent given by the MoBa participants does not allow for the storage of individual level data in any repositories or journals. Researchers who request access to MoBa data sets should apply through www.helsedata.no. Access to data sets requires approval from The Regional Committee for Medical and Health Research Ethics in Norway and a formal agreement with MoBa.
